# SnoRNPs, ZNHIT proteins and the R2TP pathway

**DOI:** 10.18632/oncotarget.6388

**Published:** 2015-11-25

**Authors:** Céline Verheggen, Bérengère Pradet-Balade, Edouard Bertrand

**Affiliations:** Institut de Génétique Moléculaire de Montpellier, Université de Montpellier, Montpellier, France

**Keywords:** Hsp90, R2TP, chaperones, snoRNP, ZNHIT proteins

HSP90 and its R2TP co-chaperone play central roles in building machineries important for RNA and DNA metabolism (see (1) for a review). These include the nuclear RNA polymerases, complexes containing PIKKs (mTOR, ATM/ATR, DNA-PK, SMG1, and TRRAP), as well as a number of ribonucleoprotein particles, such as the telomerase RNP, the spliceosomal U4 snRNA and the snoRNPs, which are essential to produce ribosomes. Given the known functions of these machineries in gene expression, protein synthesis, and DNA maintenance, it has been hypothesized that the R2TP co-chaperone carries some of the oncogenic functions of HSP90 [1]. In agreement, two R2TP components, the essential and related AAA+ ATPases RUVBL1 and RUVBL2, are overexpressed in hepatocarcinomas and colorectal cancers, and are also necessary for tumorigenesis in mouse cancer models [2]. Yet, RUVBL1 and RUVBL2 are associated to several other cellular complexes and it has not been formally demonstrated that their oncogenic activity is related to their function within the R2TP chaperone.

**Figure 1:**
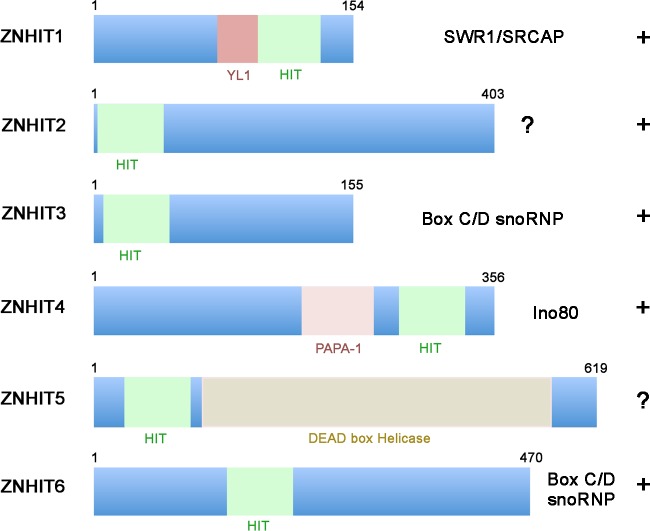
Schematic representation of the 6 different ZNHIT proteins found in human, with indication of the HIT (green) and other domains Most ZNHIT proteins bind to components of cellular machineries in which RUVBL1/2 appear to play a key role, either as an assembly cofactor (box C/D snoRNPs) or as an integral part of the purified complex (Ino80, SWR1), as indicated.

How the R2TP assists HSP90 in the assembly of protein complexes is still poorly understood. We and others took advantage of the box C/D snoRNPs, the R2TP smallest substrate, to decipher the mechanisms involved. To form a functional particle, box C/D snoRNAs have to be assembled with four core proteins: 15.5K, NOP58, NOP56 and Fibrillarin. In eukaryotes, attempts to reconstitute *in vitro* such a particle from isolated components have been so far unsuccessful. Thus, we studied the C/D snoRNP assembly pathway *in vivo*, by performing quantitative proteomic experiments using a variety of snoRNP proteins and assembly factors as baits. Importantly, we characterized a protein-only complex that preassembles 15.5K and NOP58 in the absence of snoRNA [3]. This complex contains the assembly factors NUFIP, ZNHIT3 and ZNHIT6 (also called BCD1 - see Figure 1). The key RUVBL1 and RUVBL2 ATPases were present in this complex but, surprisingly, not the other components of the R2TP chaperone: PIH1D1, RPAP3 and their associated prefoldins.

To further decipher the mechanism of box C/D snoRNP assembly, we dissected the interactions between substrates and co-factors by yeast two-hybrid assays and *in vitro* reconstitution experiments. This revealed that NUFIP forms a stable heterodimer with ZNHIT3 and interacts with the core protein 15.5K [3, 4]. Structural studies further suggested that NUFIP binding to 15.5K prevents premature activation of the catalytic activity of snoRNPs during their biogenesis [3]. One exciting hypothesis that would reconcile currently available data is that the role of the R2TP complex would be to load the essential RUVBL1/2 ATPase on the C/D core proteins NOP58 and 15.5K, thereby holding them together before their incorporation into the nascent snoRNP. In agreement with this hypothesis, RUVBL1/2 make mutually exclusive, ATP-dependent contacts with R2TP components and C/D core proteins: they bind 15.5K when loaded with ATP, and PIH1D1/RPAP3 otherwise [5, 6]. NUFIP/ZNHIT3 and ZNHIT6 could further stabilize the RUVBL1/2:NOP58:15.5K complex. Accordingly, ZNHIT6 also make ATP-dependent contacts with RUVBL1/2.

RUVBL1/2 are essential proteins that form heterohexamers or hetero-dodecamers. While these AAA+ ATPases are present in many seemingly unrelated complexes, one unifying possibility would be that some of these complexes are in fact clients of the R2TP chaperone, on which RUVBL1/2 have been loaded in order to stabilize them. In this regard, it is interesting to note that HIT-finger proteins appear to have evolved specific links with RUVBL1/2. There are six such proteins in the human genome (ZNHIT1 to ZNHIT6, Figure 1). ZNHIT3 and ZNHIT6 are associated with RUVBL1/2 during box C/D snoRNP biogenesis. ZNHIT1 and ZNHIT4, as well as RUVBL1/2, are key part of the chromatin remodeling complexes SRCAP and INO80, respectively. Remarkably, recent structural data of the yeast INO80 complex indicates that the ortholog of ZNHIT4, Ies2p, makes direct physical contacts with RuvBL1/2 and plays a central role in connecting them to the rest of the complex [7]. The two remaining HIT-finger proteins, ZNHIT2 and ZNHIT5/DDX59, have not yet been characterized. Yet, ZNHIT2 has already been found to be associated with RUVBL1/2 and RPAP3 proteins in high-throughput proteomic assays. Altogether, these data suggest that HIT-finger proteins are key partners of RUVBL1/2, possibly in relation with R2TP. They may regulate their activity or contribute to their substrate specificity (Figure 1).
